# Perioperative risk factors of psychological distress in patients undergoing treatment for esophageal cancer

**DOI:** 10.1186/s12957-020-02092-3

**Published:** 2020-12-09

**Authors:** Yu Ohkura, Junichi Shindoh, Kanako Ichikura, Harushi Udagawa, Masaki Ueno, Eisuke Matsushima

**Affiliations:** 1 Section Division of Liaison Psychiatry and Palliative Medicine, Graduate School of Medical and Dental Sciences, Tokyo Medical and Dental University, Tokyo, Japan; 2 Department of Gastroenterological Surgery, Toranomon Hospital, 2-2-2 Toranomon, Minato-ku, Tokyo, 105-8470 Japan; 3 Okinaka Memorial Institute for Medical Research, Tokyo, Japan; 4 Department of Health Science School of Allied Health Sciences, Kitasato University, Tokyo, Japan

**Keywords:** Predictive factors, Psychological distress, Coping, Health-related quality of life, Esophageal cancer

## Abstract

**Background:**

Esophageal cancer patients often feel depressed and are fearful of metastasis and death. The objective of this study was to clarify the characteristics of patients with psychological distress at all 5 time points compared with patients with no psychological distress especially from standpoints of personal coping styles and QOL.

**Methods:**

In total, 102 of 152 consecutive patients who attended the outpatient clinic at Toranomon Hospital between April 2017 and April 2019 met eligibility criteria for inclusion in this study. Questionnaires designed to identify psychological distress (HADS-scores) and assess QOL (EORTC QLQ C-30/OES18) were administered at 5 time points from the time of the first outpatient consultation to 3 months after esophagectomy. The questionnaire of coping strategies (MAC-scales) was administered at only time 1 point.

**Results:**

Based on the trends of HADS-scores, we defined two groups: “persistent high-HAD scores” and “persistent low-HADS scores.” There are strong relationships between psychological distress and coping strategy, and psychological distress and QOL. The possibility that there are relationships between stress coping strategies and some QOL status depending on some point of treatment.

**Conclusions:**

The psychological distress during the treatment course of esophageal cancer is significantly associated with the coping strategies and QOL influenced by esophagectomy. This study can provide baseline information for identifying patients in need of psychological management and paves the way for larger clinical studies in the future.

## Introduction

Esophagectomy is the mainstay of curative treatment for esophageal cancer. However, it is a highly invasive surgery with serious postoperative complications and is one of the most complex interventions in gastrointestinal surgery. Recent advances in surgical techniques and perioperative intensive care have reduced the mortality and complications associated with esophagectomy [1], but it continues to be a challenging procedure with a reported mortality rate of 2.9–3.0% and a postoperative complication rate of 42.8–50.0% [2–4]. To meet the increasing demands of healthcare, environmental safety, and food quality, various methods and techniques have been reported as reviewers’ comments. Among these, not only the diagnosis and treatment of cancer but also the mental health of the patient itself is considered to be very important factors. Previous cross-sectional studies addressing emotional outcomes after resection for esophageal cancer have suggested that in 1-year survivors, 64% felt depressed and 80% expressed fear of death and metastasis [5]. These feelings may lead to discontinuation of additional chemotherapy or chemo-radiotherapy after surgery. Moreover, a recent study identified a link between psychological state of anxiety or depression and an increased risk of death from cancer [6, 7]. We described the relationship between psychological distress and health-related quality of life (HRQOL) on each point of the treatment of esophageal cancer in the past report [8]. In this past study, we analyzed risk factors of psychological distress “on each point of the treatment”. However, when we pay attention to the chronological change of the Hospital Anxiety and Depression Scale (HADS) scores of each patient, it revealed that each patient had various mental agitation on each point and presented changes of HADS scores. The perioperative psychological stresses are usually influenced by postoperative course, physical conditions, and surrounding environment. Among these study subjects, two groups without change of the HADS scores exist during all points of the treatment. In other words, there are two types of patients who always feel stress and who always don’t feel stress during the perioperative period [9]. This fact may suggest that these two patients groups (persistent high group and persistent low group) compared with other groups may have different coping styles and that it would be of great help to select these types of patients before starting treatment for the selection of candidates of efficient intervention. Therefore, we extracted these two groups in the present study as “persistent high (H-) group” and “persistent low (L-) group” and further adding the Mental Adjustment to Cancer scale (MAC) to our previously reported scores. If the characteristics of these two patient groups could be predicted, it would be possible to develop appropriate supportive interventions and reduce the psychological stress associated with esophageal cancer. The aim of this study was to clarify the patient characteristics presenting with psychological distress after esophagectomy compared with those without significant psychological distress at all 5 time points especially from the standpoints of personal coping styles and HRQOL.

## Methods

### Definition of psychological distress

The definition of psychological distress is a negative emotional state characterized by physical and/or emotional discomfort, pain, or anguish. In other words, it is psychological discomfort that interferes with your activities of daily living. The definition of “psychological distress” and the definition of “anxiety and depression” are not the same concepts. However, psychological distress can result in negative views of the environment, others, and the self. Sadness, anxiety, distraction, and symptoms of mental illness are manifestations of psychological distress [10, 11].

Several screening scales have been developed for the early detection of patients’ psychological distress. The HADS score was developed by Zigmond and Snaith [12] and is well accepted to screen for psychiatric problems in medically ill patients. Generally, cancer patients whose psychological distress is mainly characterized by anxiety and depression can benefit from using HADS to detect this distress. And also, Kugaya et al. [9] proved the reliability and validity of the Japanese version of HADS, and the scale appeared to be simple, sensitive, and specific for screening for psychological distress in Japanese cancer patients. Therefore, we use HADS for screening for psychological distress in the present study.

### Study design and endpoints

Participants comprised 152 consecutive patients who attended the outpatient clinic at the Department of Gastrointestinal Surgery at Toranomon Hospital between April 2017 and April 2019 were assessed for trial eligibility. Among these 152 patients, 102 patients who met the eligibility criteria participated in this study. The inclusion criteria were as follows: esophageal cancer including Siewert type I/II tumors of the esophagogastric junction; scheduled for subtotal esophagectomy; age 20 to 85 years; performance status 0 to 2; ability to provide informed consent; with previous oncological therapies for cancer; with previous surgical treatment for other diseases. We included patients 20–85 years old. The reason is because patients may have senile dementia. Generally, about 3% of people between the ages of 65–74 have dementia, 19% between 75 and 84, and nearly half of those over 85 years of age [13]. Therefore, we selected the patients 20–85 years old.

**Fig. 1:**
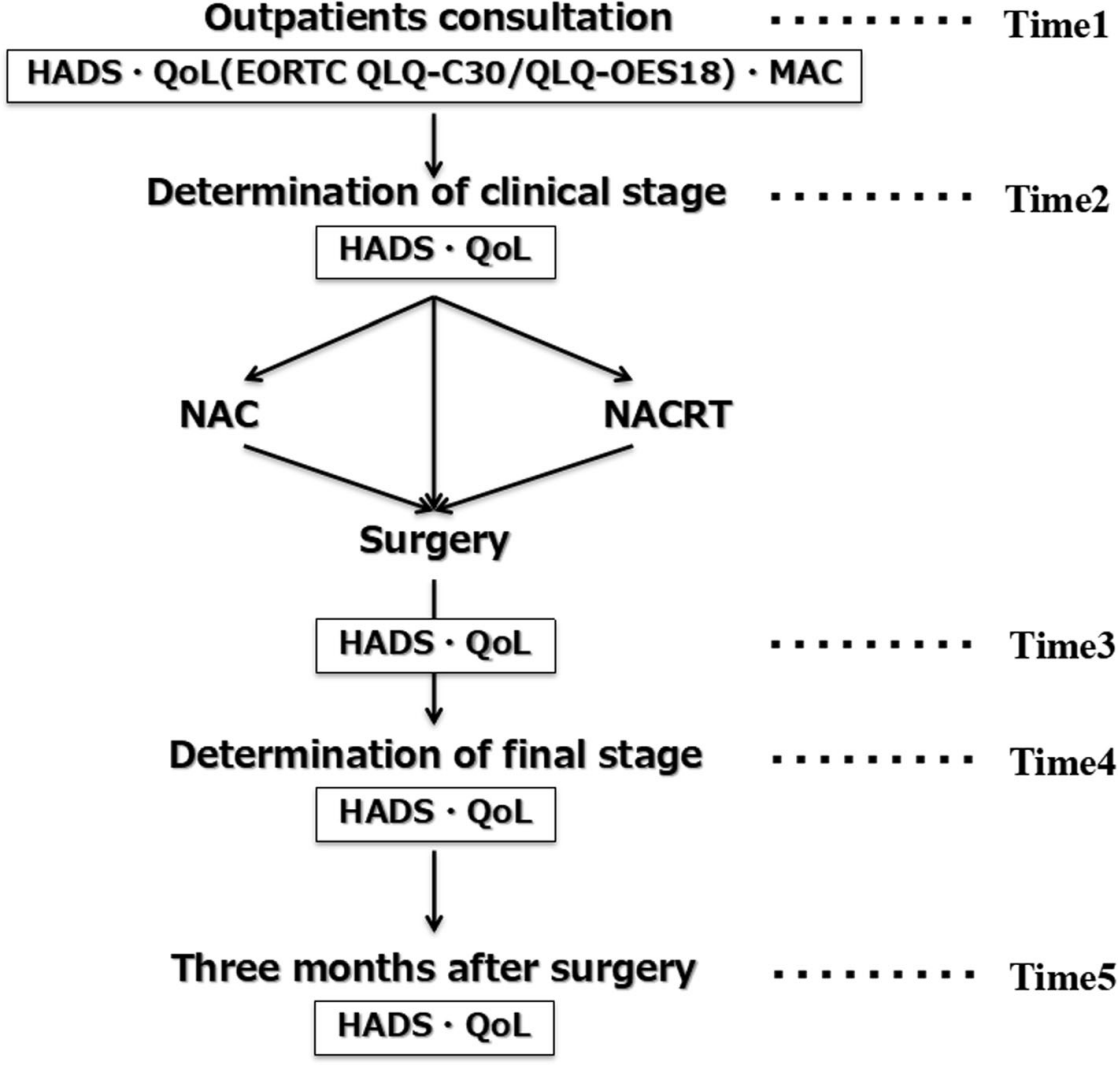
Flow of patients through the study. Time 1, before definitive diagnosis; time 2, after determination of clinical stage; time 3, postoperatively before final staging; time 4, determination of final stage at 1 month after esophagectomy; time 5, 3 months after esophagectomy

**Fig. 2:**
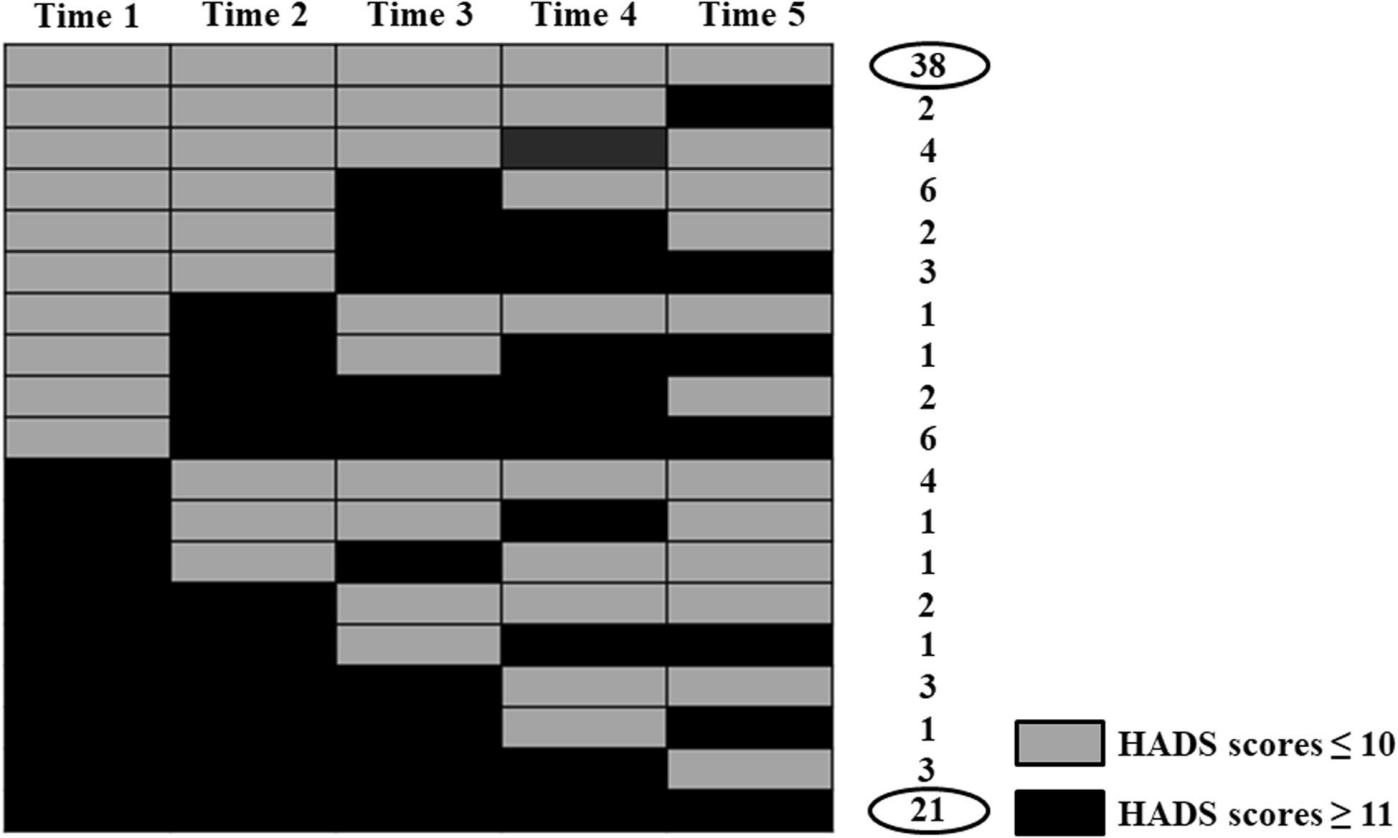
The trend of the HADS scores at time 1 to time 5. Twenty-one patients were allocated to the “persistent high (H-) group,” and 38 patients were allocated to the “persistent low (L-) group”

The following exclusion criteria were applied: informed consent has not been obtained; on treatment for mental disorder; unsuitability for participation in the study because of psychological or physical stress in the opinion of the medical staff; refusal to undergo surgery; and requested nonsurgical treatment. The flow diagram of this study is shown in Fig. 1. Questionnaires were administered at 5 time points as follows: at the time of outpatient consultation before definitive diagnosis (time 1); at the time of determination of clinical stage before treatment; both of surgery and neoadjuvant therapy (time 2); about 2 weeks after surgery before final staging (time 3); at the time of determination of final staging (time 4); and at 3 months after surgery (time 5). The questionnaires were administered in a fixed waiting room at our institution because we would like to perform the survey acquisition under the stable condition as much as possible. We selected three questionnaires; the Japanese version of HADS, the MAC scale, and the European Organization for Research and Treatment of Cancer Quality of Life Questionnaire Core 30/the oesophageal cancer-specific module (EORTC QLQ-C30/OES18). The questionnaires (HADS/EORTC QLQ-C30/OES18) were administered at all 5 time points, and the MAC scale was administered once, at time 1. From the trends of HADS scores on each point of the treatment, we selected two groups: “persistent high HADS scores” and “persistent low HADS scores”. Of 102 patients who met the eligibility criteria and participated in this study, 21 patients were allocated to the “persistent high (H-) group,” and 38 patients were allocated to the “persistent low (L-) group” (Fig. 2). We investigated the characteristics of the H-group compared with the L-group especially from standpoints of personal coping styles and HRQOL. Esophageal cancer staging (clinical (c-)/pathological(p-) stage) is defined by the UICC TNM grading system, 7th edition; the sub-classifications based on the depth of invasion of the primary tumor (c-/p-T factors), lymph node involvement (c-/p-N factors), and extent of metastatic disease (c-/p-M factors) [14]. T factors classified the following; Tis: high-grade dysplasia, T1: invasion into the lamina propria, muscularis mucosae, or submucosa, T2: invasion into the muscularis propria, T3: invasion into the adventitia, T4a: invades resectable adjacent structures (pleura, pericardium, diaphragm), and T4b: invades unresectable adjacent structures (aorta, vertebral body, trachea). Similarly, N factors classified the following: N0: no regional lymph node metastases, N1: 1 to 2 positive regional lymph nodes, N2: 3 to 6 positive regional lymph nodes, and N3: 7 or more positive regional lymph nodes. M factors classified the following: M0: no distant metastases and M1: distant metastases [14]. All postoperative complications were graded using the Clavien-Dindo classification [15]; events more severe than grade ≥ III were recorded as complications. We chose this cut-off value on the basis of the Japanese version of HADS, which has been validated for Japanese patients with cancer [12]; a cut-off HADS total score ≥ 11 has been recommended for identifying patients with potential adjustment disorder and major depression [12]. We defined a total score ≥ 11 as indicating psychological distress. The study protocol was approved by the institutional review boards of the Graduate School of Medical and Dental Sciences (approval number M2016-241) and Toranomon Hospital (approval number 1312) and registered with the UMIN Clinical Trials Registry (UMIN-CTR, R000033229). All procedures were conducted in accordance with the ethical standards of the Helsinki Declaration of 1975. Informed consent was obtained from all study participants at the time of the first outpatient appointment.

### Measures

#### Mental adjustment to cancer scale

This scale (MAC scale) assesses the extent to which patients respond and adjust to their diagnosis of cancer and its treatment [16, 17]. It was developed as a self-rating questionnaire that would be patient-friendly and could be administered easily at busy oncology clinics. The scale includes five subscales that measure five types of response: fighting spirit (“I firmly believe that I will get better,” 16 items); helpless/hopelessness (‘I feel that life is hopeless,” 6 items); anxious preoccupation (“I suffer great anxiety about it,” 9 items); fatalism (“I’ve left it all to my doctors”, 8 items); and avoidance (“I don’t really believe I have cancer,” 1 item). Each item is scored on a 4-point Likert scale (1, “definitely does not apply to me”; 4, “definitely applies to me”). Scores for the subscales are calculated by summing the answers for the assigned items. Generally, the coping skills do not greatly change in the short time; therefore, we did not measure the MAC scale at each time point [18, 19]. This questionnaire was administered once, at time 1, in this study.

#### Hospital Anxiety and Depression Scale

This scale (HADS) is a 14-item self-report questionnaire comprising of two subscales measuring symptoms of depression (HADS-D) and symptoms of anxiety (HADS-A) over the last week. Each subscale contains 7 items with scores ranging from 0 to 21 [11]. A total score of ≥ 11 on either subscale indicates a definitive case. The Japanese version of HADS was back-translated by Kitamura [20] and its reliability and validity were confirmed by Kugaya et al. [9]. The questionnaire was administered at all 5 time points in this study.

#### The European Organization for Research and Treatment of Cancer Quality of Life Questionnaire Core 30

This questionnaire (EORTC QLQ-C30) is one of the most frequently used questionnaires to measure health-related quality of life in patients with cancer [21–23]. Its 30 question items have been validated in several studies in various types of cancer. This score is a 30-item questionnaire divided into five functional scales (physical, role, cognitive, emotional, and social), three symptom scales (fatigue, pain, and nausea/vomiting), six single-item questions (financial impact and various physical symptoms such as dyspnea, insomnia, anorexia, constipation, and diarrhea), and a global health status/quality of life. The items on the functional subscales are rated on a 4-point Likert scale (1, “not at all”; 4, “very much”). The raw subscale scores are transformed into a scale of 0–100 (0, poor QOL; 100, excellent QOL). The questionnaire was administered at all 5 time points in this study.

#### EORTC QLQ-OES18 questionnaire

The EORTC QLQ-OES18 is a self-reported 18-item questionnaire designed to assess health-related quality of life in patients undergoing a single treatment or a combination of treatments for esophageal cancer (i.e., esophagectomy, chemotherapy, radiotherapy, and/or chemo-radiotherapy). It includes 12 items grouped into 4 symptom scales (dysphagia; 3 items, eating; 4 items, reflux; 2 items, and pain; 3 items) and 6 single items (trouble with swallowing saliva, choking, dry mouth, taste, cough, and speech). The time frame is “during the past week.” All items are scored using a 4-point Likert scale (1, “not at all”; 4, “very much”) and responses to the questionnaires were transformed into a 0–100 scale using EORTC guidelines [24]. The questionnaire was administered at all 5 time points in this study.

### Operative procedure for esophagectomy

We perform esophagectomy with two-field or three-field lymphadenectomy depending on the degree of disease progression and the surgical risk involved. The operative thoracic approach entails video-assisted thoracoscopic surgery or thoracotomy and the abdominal approach involves hand-assisted laparoscopic surgery or open laparotomy depending on the individual case. We generally preserve the thoracic duct in patients with clinical stage I disease but resect it in those with clinical stage ≥ II disease for the purpose of lymphadenectomy [13]. However, we resected the thoracic duct even if the patient has clinical stage I disease when we suspect lymph node metastasis or have confirmed a metastasis in the lymph nodes along the bilateral recurrent laryngeal nerves on intraoperative pathologic analysis. However, we try to preserve the thoracic duct in patients at high risk of impaired hepatic or pulmonary function. A manually sutured esophagogastric or esophagoileal anastomosis in the neck is fashioned for all patients [25–29].

### Statistical analysis

Differences between the two groups (H-group/ L-group) were tested for statistical significance using Fisher’s exact test, the unpaired Student’s *t* test, the Mann-Whitney *U* test, and Pearson’s chi-squared test as appropriate.

Risk factors for psychological distress (H-group) were assessed by bivariate logistic regression analysis (Backward stepwise selection). The variables with a *p* value less than 0.05 in univariate analysis were entered into bivariate logistic analysis. Odds ratios (ORs) and their 95% confidence intervals (CIs) were calculated. A *p* value less than 0.05 was considered statistically significant in bivariate logistic analysis. The correlations between the MAC scale and HRQOL status were assessed using Spearman’s correlation coefficient and statistical significance was tested using Spearman’s rank-sum test. The internal consistency (reliability) of each scale was estimated by Cronbach's alpha coeffcient [30]. A value of 0.70 or greater was considered acceptable for group comparison. All analysis was performed using SPSS for Windows software (version 19.0 J; IBM Corp., Armonk, NY).

## Results

### Patient characteristics

Fifty of the 152 patents considered for participation in the study were excluded because they had incomplete data (*n* = 27), declined to participate (*n* = 11), did not undergo esophagectomy (*n* = 11), or were receiving treatment for a psychiatric disorder (*n* = 1), leaving 102 patients for inclusion in the study. From the trends of HADS scores on each point of the treatment of esophageal cancer (Fig. 2), we selected two groups: “persistent high HADS scores” and “persistent low HADS scores”. The characteristics of these patients in the two groups are shown in Table 1.

**Table 1:** Patient demographics and clinical characteristics

Variables	Persistent low group (***N*** = 38)	Persistent high group (***N*** = 21)	Others (***N*** = 43)	***p*** value
**Age, years, mean ± SD**	64.5 ± 8.079	65.5 ± 6.875	69.2 ± 9.398	0.680
**Male sex, %**	86.8	85.7	81.4	0.904
**BMI, kg/m**^**2**^**, median**	22.7 (17.4–27.7)	20.1 (14.1–41.9)	22.0 (15.0–32.1)	0.024
**History of cancer**				0.243
Yes	6	6	12	
No	32	15	31	
**History of surgery**				0.193
Yes	10	9	17	
No	28	12	26	
**History of alcohol consumption**				0.353
Yes	34	18	39	
No	4	3	4	
**History of smoking**				0.953
Yes	31	17	39	
No	7	4	4	
**Brinkman index**	632 (0–2700)	600 (0–2760)	741 (0–3040)	0.975
< 600				0.544
≥ 600	15	10	19	
	23	11	24	
**Thoracic approach**				0.234
VATS	35	17	36	
Open	1	3	5	
None: transhiatal	2	1	2	
**Abdominal approach**				0.022
HALS	13	11	23	
Open	6	7	11	
Laparoscopic	19	3	9	
**Lymphadenectomy**				0.247
D0	1	2	1	
D2/3	37 (11/26)	19 (5/14)	42 (13/29)	
**Curability**				0.665
R0	37	20	39	
R1/2	1	1	4	
**Reconstruction**				0.780
Gastric tube	25	12	29	
Ileocolic	8	6	12	
Other	5	3	2	
**Thoracic duct**				0.012
Resected	16	16	30	
Preserved	22	5	13	
**Reconstruction route**				0.258
Retrosternal	30	19	39	
Posterior mediastinal	8	2	4	
**Preoperative treatment**				0.160
Yes	16	5	27	
No	22	16	16	
**Operation time (min)**	592 (350–734)	597 (415–703)	574 (213–774)	0.795
**Blood loss (mL)**	130 (25–1175)	274 (25–1378)	260 (38–1094)	0.067
**G3 postoperative complications**				0.855
Yes	8	4	11	
No	30	17	32	
**cT factor (7th)**				0.303
1a/1b	2/16	1/5	2/10	
2	12	6	7	
3	6	6	18	
4a/4b	0/2	2/1	2/4	
**cN factor (7th)**				
0	25	7	13	0.029
1	10	11	15	
2	3	1	15	
3	0	2	0	
**cStage (7th)**				0.109
I (IA, IB)	12/9	5/0	10/2	
II (IIA, IIB)	3/7	1/7	1/5	
III (IIIA, IIIB, IIIC)	3/1/1	1/1/4	9/7/5	
IV	2	2	4	
**p T factor (7th)**				0.126
0	2	2	2	
1a/1b	5/19	1/6	3/8	
2	8	3	9	
3	2	6	19	
4a/4b	1/1	2/1	2/0	
**p N factor (7th)**				0.276
0	26	10	13	
1	8	8	12	
2	3	1	11	
3	1	2	7	
**p Stage (7th)**				0.062
0	2	1	0	
I (IA, IB)	18/2	6/0	8/3	
II (IIA, IIB)	2/8	0/3	1/5	
III (IIIA, IIIB, IIIC)	4/0/1	3/1/5	12/7/5	
IV	1	2	2	
**Location of tumor**				0.510
Cervical Esophagus	2	1	1	
Upper thoracic esophagus	5	4	10	
Middle thoracic esophagus	15	12	19	
Lower thoracic esophagus	7	3	10	
Abdominal esophagus	1	0	0	
Esophagogastric junction	8	1	3	
**MAC scale**				
Fighting spirit	51.0 (34–59)	44.0 (27–57)	48.0 (29–60)	0.001
Helpless/hopeless	7.0 (6–16)	12.0 (6–24)	10.0 (6–16)	< 0.001
Anxious preoccupation	21.0 (13–29)	25.0 (17–31)	23.0 (14–32)	< 0.001
Fatalism	18.0 (8–29)	21 (12–30)	22.0 (13–28)	0.008
Avoidance	1.0 (1–4)	1.0 (1–4)	2.0 (1–4)	0.793

*Abbreviations*: *SD*, standard deviation; *BMI*, body mass index; *EORTC*, European Organization for Research and Treatment; *HADS*, Hospital Anxiety and Depression Scale; *VATS*, video-assisted thoracoscopic surgery; *HALS*, hand-assisted laparoscopic surgery; *MAC*, mental adjustment to cancer; *VATS*, video-assisted thoracoscopic surgery

The mean age of L-group/H-group was 65.3/67.0 years, 86.8%/85.7% were male, and median body mass index was 22.7/20.1, respectively. Univariate analysis between the two groups showed a significant difference in the abdominal approach (*p* = 0.022), resection or preservation of the thoracic duct (*p* = 0.012), clinical N factors (*p* = 0.029), and MAC scales (fighting spirits; *p* = 0.001, helpless/hopeless; *p* < 0.001, anxious preoccupation; p < 0.001, and fatalism; *p* = 0.008).

### EORTC QLQ C-30 and psychological distress

**Fig. 3:**
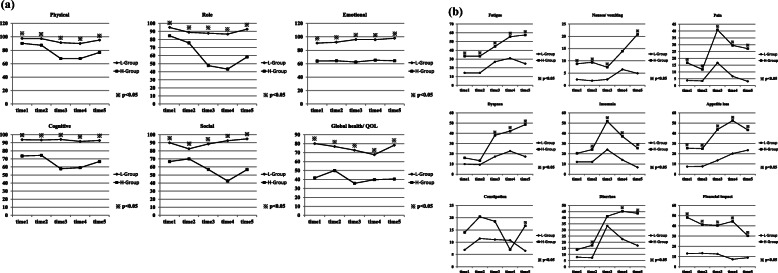
The trend of the score of EORTC QLQ C-30. a Functioning scales: physical functioning, role functioning, emotional functioning, cognitive functioning, social functioning, and global health status/quality of life. b Symptom scales: fatigue, pain, nausea/vomiting, pain, dyspnea, insomnia, anorexia, constipation, diarrhea, and financial impact

The trend of the scores of EORTC QLQ C-30 on each 5 points of the treatment of esophageal cancer is recorded in Fig. 3 and Table 2; data were compared between the two groups (H-group/L-group). Figure 3 a shows five functional subscales such as physical functioning, role functioning, emotional functioning, cognitive functioning, social functioning, and global health status/quality of life. In almost all the functional subscales on each 5 points, the H-group had a significantly lower and unhealthier level of functioning than the L-group. Figure 3 b shows 9 symptom scales such as fatigue, pain, nausea/ vomiting, pain, dyspnea, insomnia, anorexia, constipation, diarrhea, and financial impact. In almost all the symptom scales on each 5 points, the H-group had a significantly higher level of symptomatology or problems than the L-group.

**Table 2:** The details of the QOL status: EORTC QLQ C-30 and EORTC OES-18

	Time 1			Time 2			Time 3			Time 4			Time 5		
	H	L	P	H	L	P	H	L	P	H	L	P	H	L	P
**QLQ C-30**															
**Physical**	90.3	97.6	0.001	87.5	97.2	0.001	67.6	91.3	< 0.001	67.6	90.0	< 0.001	76.7	95.2	< 0.001
**Role**	84.6	95.0	0.017	75.8	88.9	0.011	47.6	87.7	< 0.001	43.3	86.7	< 0.001	58.3	92.8	< 0.001
**Emotional**	63.9	90.6	< 0.001	64.3	92.0	< 0.001	62.5	96.0	< 0.001	65.3	95.7	< 0.001	64.3	97.9	< 0.001
**Cognitive**	73.6	93.8	< 0.001	74.4	93.4	< 0.001	57.8	94.1	< 0.001	59.1	91.7	< 0.001	66.7	92.8	< 0.001
**Social**	66.7	90.0	0.001	70.0	82.5	0.045	56.9	88.5	< 0.001	42.6	92.6	< 0.001	56.7	94.8	< 0.001
**Global health**	41.7	80.1	< 0.001	50.0	76.8	0.002	35.7	72.7	< 0.001	39.8	67.7	< 0.001	40.5	78.3	< 0.001
**Fatigue**	33.3	14.2	0.001	33.3	14.2	< 0.001	44.4	26.9	< 0.001	55.6	31.0	< 0.001	57.4	24.7	< 0.001
**Nausea**	8.8	2.4	0.014	9.4	1.9	0.006	7.4	2.4	0.040	13.9	6.5	0.132	20.8	4.9	0.005
**Pain**	16.7	3.8	0.003	11.9	3.4	0.017	40.9	16.7	< 0.001	29.5	6.8	< 0.001	27.1	3.1	< 0.001
**Dyspnea**	15.9	9.9	0.187	13.3	9.3	0.383	38.1	17.2	0.003	42.2	22.6	0.017	48.5	17.2	< 0.001
**Insomnia**	20.4	12.0	0.182	24.1	12.0	0.034	51.9	24.0	0.001	37.0	14.0	< 0.001	25.6	6.7	0.002
**Appetite loss**	25.5	7.4	0.002	25.0	7.6	0.003	43.7	13.5	0.001	52.4	20.2	0.001	43.3	23.5	0.032
**Constipation**	14.0	6.9	0.149	20.4	11.5	0.140	18.5	11.1	0.212	7.0	10.8	0.418	16.7	6.5	0.047
**Diarrhea**	14.0	7.9	0.182	17.5	7.4	0.031	41.2	33.3	0.389	45.2	22.6	0.015	43.6	17.3	0.002
**Financial**	48.3	13.0	< 0.001	41.2	13.3	< 0.001	40.5	12.5	0.001	44.4	7.3	< 0.001	30.3	8.9	0.003
**OES 18**															
**Dysphagia**	63.5	94.8	0.003	82.2	96.1	0.002	62.5	92.6	< 0.001	64.4	84.8	< 0.001	63.5	89.9	0.328
**Eating**	58.3	93.5	< 0.001	68.3	94.3	0.001	59.3	83.9	< 0.001	41.7	74.3	< 0.001	47.2	78.8	0.010
**Reflux**	87.5	96.2	0.004	87.8	96.2	0.010	86.1	94.6	0.040	81.7	90.0	0.122	75.9	91.0	0.161
**Eso. pain**	82.7	98.0	< 0.001	87.5	97.4	0.005	71.4	93.8	0.001	75.9	94.0	0.005	74.1	97.5	0.010
**Saliva**	90.0	97.4	0.033	90.5	100	0.026	80.4	95.4	0.002	74.5	91.7	0.002	91.1	96.8	0.442
**Choking**	86.7	96.5	0.011	88.3	97.4	0.012	74.5	90.7	0.003	70.6	88.9	0.001	73.8	89.2	0.105
**Dry mouth**	70.8	88.0	0.009	70.8	85.2	0.031	58.8	79.3	0.007	57.1	86.0	0.002	69.7	92.5	0.442
**Taste**	87.0	95.4	0.073	76.2	91.7	0.050	66.7	88.6	< 0.001	61.5	89.6	0.001	69.4	89.7	0.161
**Cough**	90.0	94.6	0.249	86.0	94.6	0.053	64.1	78.1	0.092	53.3	79.0	0.009	60.6	85.1	0.721
**Speech**	91.7	94.4	0.469	95.0	94.4	0.899	62.2	81.2	0.010	47.6	72.6	0.004	56.7	92.2	0.721

### EORTC QLQ OES-18 and psychological distress

**Fig. 4:**
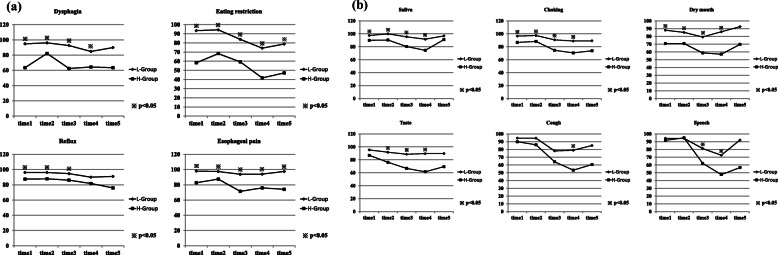
The trend of the score of EORTC QLQ OES18. **a** Symptom scales: dysphagia, eating restriction, reflux, esophageal pain. **b** Single items: trouble with swallowing saliva, choking, dry mouth, taste, cough, and speech

The trend of the scores of EORTC QLQ OES-18 on each 5 points is recorded in Fig. 4 and Table 2; data were compared between the two groups (H-group/L-group). Figure 4 a and b show four symptom scales such as dysphagia, eating restriction, reflux, esophageal pain, and 6 single items such as trouble with swallowing saliva, choking, dry mouth, taste, cough, and speech. In almost all these scales on each 5 points, the H-group had a significantly higher level of symptomatology or problems than the L-group.

### The relations between the stress coping strategies and QOL status

There were correlations between the MAC scale and QOL status in some scales. The relationship between two variables is generally considered moderate when their correlation coefficient (*r*) value is larger than 0.5 and also considered strong correlation when their *r* value is larger than 0.7 [31]. The internal consistency (reliability) of each scale was estimated by Cronbach’s alpha coefficient. A value of 0.70 or greater was considered acceptable for group comparison [30].

In this study, the *r* values and Cronbach’s alpha coefficient between the MAC scale (helpless/hopeless) and some variables of EORTC QLQ C-30/OES18 in several points of perioperative treatment were global (time 3; *r* = − 0.521 (*α* = 0.501); *p* < 0.001/time 5; *r* = − 0.576 (α = 0.558) ; *p* < 0.001), physical (time 5; *r* = − 0.628 (α = 0.614) ; *p* < 0.001), emotional (time 1; *r* = − 0.520 (α = 0.537) ; *p* < 0.001/time 3; *r* = − 0.559 (*α* = 0.560) ; *p* < 0.001), cognitive (time 3; *r* = − 0.558 (α = 0.533) ; *p* < 0.001/time 4; *r* = − 0.524 (*α* = 0.522) ; *p* < 0.001), social (time 5; *r* = − 0.542 (α = 0.692) ; *p* < 0.001), fatigue (time 2; *r* = 0.530 (α = 0.533) ; *p* < 0.001/time 5; *r* = 0.583 (α = 0.562) ; *p* < 0.001), financial difficulty (time 4; *r* = 0.509 (*α* = 0.516) ; *p* < 0.001), OES18: eating restriction (time 3; *r* = − 0.518 (α = 0.516) ; *p* < 0.001/ time 4; *r* = − 0.501 (α = 0.733) ; *p* = 0.001/ time 5; *r* = − 0.573 (α = 0.589) ; *p* < 0.001), and speech (time 5; *r* = 0.503 (*α* = 0.770) ; *p* = 0.001). Among these variables, there were moderate correlations between helpless/hopeless and eating restriction scale at time 4/ speech scale at time 5 considering Cronbach's alpha coefficient. On the other hand, there was a strong correlation between helpless/hopeless and role functioning scale at time 5 (*r* = − 0.752 (*α* = 0.752); *p* < 0.001).

## Discussion

In this study, we evaluated psychological distress, stress coping strategy, and quality of life in patients with esophageal cancer from the time of the outpatient consultation (time 1) through to 3 months after esophagectomy (time 5). Our results showed that there are strong relationships between psychological distress and stress coping strategy, and psychological distress and quality of life. And also, this study suggests that there are relationships between stress coping strategies and some HRQOL status depending on the point of treatment.

We discussed and described that the clinical factors associated with psychological distress, focusing on the influence of HRQOL and the coping strategies of all patients with esophageal cancer on each point of the treatment [8]. In our past study, we analyzed risk factors of psychological distress “on each point of the treatment.” However, when we pay attention to the chronological change of HADS scores of each patient, it revealed that each patient had various mental agitation on each point and presented changes of HADS scores, for example, 21 patients had “persistent high HADS scores”, 38 patients had “persistent low HADS”, 12 patinets had “High-Low”, 11 patients had “Low-High”, 15 patients had “Low-High-Low”, and so on [8]. In the previous cross-sectional studies, the emotional outcomes after resection for esophageal cancer have suggested that in 1-year survivors, 64 % felt depressed, and 80% expressed fear of death and metastasis [5]. Our present research revealed that about 60–70% of patients with esophageal cancer on each point of the treatment had psychological distress like the past report. However, only about 20% of patients had psychological distress at all times (persistent high HADS scores) during the treatment. Among these study subjects, two groups without change of the HADS scores exist during each point of the treatment. The possibility that these two patients group (persistent high group and persistent low group) compared with other groups may have a different coping style was guessed. Therefore, we extracted these two groups in the present study as “persistent high (H-) group” and “persistent low (L-) group” and further adding the coping skills questionnaire (MAC scale) to our previously reported scores.

### Patients background

In the H-group, there was a tendency for many cases to be resected with thoracic duct and to be positive for lymph node metastases. We generally resect the thoracic duct in patients with clinical stage ≥ II advanced disease for the purpose of lymphadenectomy. One possible explanation for these results is that the degree of disease progression may affect perioperative psychological distress.

### Coping skills

In the past reports, the possibility that the coping skill improves the psychological distress had been discussed because there were significant associations between the coping skills and psychological distress [16, 17, 32]. However, it is still unclear whether the coping skills act on the psychological distress effectively in the various situations of the episode of the esophageal cancer treatment. In the present study, patients with a helpless/hopeless response, anxious preoccupation, and fatalism were at increased risk of psychological distress, whereas those with a fighting spirit response were better able to adjust to their situations. And also, the results showed that the most beneficial response is fighting spirit and the most deleterious response may be helpless/hopeless. If we can grasp the stress coping-style of the patients with esophageal cancer even one investigation before treatment, it would be possible to develop appropriate supportive interventions and reduce the psychological stress, anxiety, and depressive feelings associated with esophageal cancer and surgery.

### HRQOL: EORTC QLQ C-30/OES18

The emotional aspects such as worry and irritability have been shown to be associated with HRQOL [33]. And also, in the past reports, there was a significant association between the HRQOL and psychological distress [34–36]. We similarly described the psychological distress during the treatment course of esophageal cancer is significantly associated with HRQOL influenced by esophagectomy [8]. In the present study, although we selected only two groups of patients with or without psychological distress at all times for the perioperative period, we were able to describe that patients with psychological distress at all times had a significantly lower and unhealthier level of functioning in almost all the functional scales, and a higher level of symptomatology or problems in almost all the symptom scales than the patients without psychological distress. The symptoms that patients with esophageal cancer have would be affected physically by esophageal cancer itself such as “Fatigue,” “Nausea/vomiting,” “Pain,” and “Appetite loss” in EORTC QLQ C-30 and “Dysphagea,” “Eating restriction,” “Reflux,” and “Esophageal pain” in OES 18 especially at time 1/time 2 (preoperation). And also, the symptoms that patients after esophagectomy have would be affected physically by surgery itself such as “Fatigue,” “Nausea/vomiting,” “Pain,” “Dyspnea,” “Insomnia,” and “Appetite loss” in EORTC QLQ C-30 and “Dysphagea,” “Eating restriction,” “Reflux,” “Esophageal pain,” “Choking,” “Dry mouth,” “Taste,” “Cough,” and “Speech” in OES 18 especially at time 3/time 4 (postoperation). Through all episodes of treatment, “The financial difficulties status,” “Role functioning,” and “Social status” was a significant factor for psychological distress. We could guess that the patients have large anxiety about treatment strategies and costs of esophageal cancer treatments particularly in the era of multimodal treatment which requires more time and cost. Also, patients feel so sorry to trouble his or her family because of their physical problems such as “eating difficulties.” Except for our past [8] and present reports, there is no report which described more detail about the association between HADS and HRQOL.

### The relations between coping skills and HRQOL

It is usually expected that patients with advanced diseases have a tendency of higher HADS scores and more impaired QOL. It is also suggested that those who were informed of very advanced or non-curable disease status revealed surgically or pathologically would have higher HADS scores and more impaired QOL after the information. Psychological distress, QOL, disease status, and its recognition by the patient, all these factors should correlate with each other and their causal relationships are complicated and can vary with time course. Patients included neither in H-group nor in L-group, who showed chronological changes of HADS score levels, might have been influenced by the information provided through the treatment course. Further examination will be necessary about these strategies in the future. However, this study showed that more than 50% of the patients consistently had high or low HADS scores throughout the course of the treatment of their esophageal cancers, and surprisingly, there was little difference in clinical characteristics between the two groups, but the two groups had a clear difference in 4 out of 5 subscales of the MAC scale measured preoperatively. Therefore, at least for such half or more patients, preoperative MAC scale evaluation can be useful in selecting patients who can benefit by appropriate supportive interventions on their coping skills and it may reduce their psychological distress and hopefully improve their QOL.

There is no report which described the association between the MAC scale and HRQOL in the past. Therefore, it is still unclear whether the Coping skills act on the HRQOL effectively in the various situations of the episode of the esophageal cancer treatment. We investigated in more detail that the association between stress coping strategy and HRQOL on each point of the treatment of esophageal cancer. As described above, most of the symptoms that patients with esophageal cancer have would be affected physically by esophageal cancer itself and the treatment including an operation. However, there is a possibility that coping skills would be related to the emergence of postoperative symptoms. In fact, there was a correlation between the MAC scale and QOL status in some scales such as helpless/hopeless—eating restriction, speech, and role functioning in the present study.

The major limitations for this study are the single-center design and the small sample size investigated. An external validation study involving a sufficient number of patients would be needed to confirm our observations; a multicenter study with a larger number of cases is also warranted. We selected four questionnaires according to the past reports, but using these questionnaires might not be sufficient to fulfill the purpose of our study. There might be other more suitable questionnaires to satisfy this study purpose. There were also several factors potentially associated with psychological distress related to treatment for esophageal cancer that could not be controlled for in this study. Nevertheless, our results confirm that the risk of psychological distress can be estimated reasonably accurately using the clinical factors investigated in this study. Prospective accumulation of clinical data using this study could provide important information for better psychological management of patients undergoing treatment for esophageal cancer.

## Conclusion

This study showed that the psychological distress during the treatment course of esophageal cancer is significantly associated with the coping strategies and HRQOL influenced by esophagectomy. The current results may warrant prospective intervention through enhanced recovery after surgery to better manage patients undergoing highly invasive procedures for esophageal cancer. This study uses basic clinical data and may provide the baseline information for risk stratification for psychological management and for future clinical studies in these patients.

## Data Availability

Some advocates of clinical data sharing are keen for data to be shared in agreed, standardized formats to facilitate its automated reuse for statistical analysis.

## Abbreviations

HADS: Hospital Anxiety and Depression Scale
MAC scale: Mental Adjustment to Cancer scale
EORTC QLQ-C30/OES18: the European Organization for Research and Treatment of Cancer Quality of Life Questionnaire Core 30/the oesophageal cancer-specific module
HRQOL: Health-related quality of life
